# Surface plasmon induced direct detection of long wavelength photons

**DOI:** 10.1038/s41467-017-01828-2

**Published:** 2017-11-21

**Authors:** Jinchao Tong, Wei Zhou, Yue Qu, Zhengji Xu, Zhiming Huang, Dao Hua Zhang

**Affiliations:** 1 School of Electrical and Electronic Engineering, Nanyang Technological University, Nanyang Avenue, 639798 Singapore, Singapore; 2 State Key Laboratory of Infrared Physics, Shanghai Institute of Technical Physics, Chinese Academy of Sciences, 500 Yu Tian Road, 200083 Shanghai, China; 3 Key Laboratory of Space Active Opto-Electronics Technology, Shanghai Institute of Technical Physics, Chinese Academy of Sciences, 500 Yu Tian Road, 200083 Shanghai, China

## Abstract

Millimeter and terahertz wave photodetectors have long been of great interest due to a wide range of applications, but they still face challenges in detection performance. Here, we propose a new strategy for the direct detection of millimeter and terahertz wave photons based on localized surface-plasmon-polariton (SPP)-induced non-equilibrium electrons in antenna-assisted subwavelength ohmic metal–semiconductor–metal (OMSM) structures. The subwavelength OMSM structure is used to convert the absorbed photons into localized SPPs, which then induce non-equilibrium electrons in the structure, while the antenna increases the number of photons coupled into the OMSM structure. When the structure is biased and illuminated, the unidirectional flow of the SPP-induced non-equilibrium electrons forms a photocurrent. The energy of the detected photons is determined by the structure rather than the band gap of the semiconductor. The detection scheme is confirmed by simulation and experimental results from the devices, made of gold and InSb, and a room temperature noise equivalent power (NEP) of 1.5 × 10^−13^ W Hz^−1/2^ is achieved.

## Abstract (web summary)

The detection of terahertz and millimeter waves has many applications, but there are still limitations in their technical performance. Here, Tong et al. demonstrate the direct detection of long-wavelength radiation through surface plasmon excitation and a corresponding improvement in detection performance.

## Introduction

It is well known that long-wavelength photodetectors, especially for the millimeter and terahertz wave ranges, have a wide range of applications in areas such as meteorology, astronomy, medicine, communication, and biology^1–4^. Conventional photodetection based on photo-excited electron–hole pairs (EHPs) in a semiconductor does not perform well for such long-wavelength photons (LWPs) due to the relatively small photon energy and strong background thermal noise.

Commercially available LWP detectors^5^ include Golay cells, pyroelectric elements, bolometers, and Schottky barrier diodes (SBDs)^6–8^. Based on thermal sensing mechanisms, the first three either suffer from a slow response (only up to a few hundred hertz modulation frequency for Golay cells and pyroelectric elements, though their noise equivalent power (NEP) can be as low as 10^−10^ W Hz^−1/2^ at room temperature^9^) or require cryogenic cooling for normal operation (4.2 K for typical Si bolometers). SBDs, widely used in radio-frequency (RF) and microwave ranges, are high-speed but require advanced fabrication and material growth techniques.

In the past decades, much effort has been devoted to developing novel techniques for LWP detection. Terahertz quantum-well infrared photodetectors, based on intersubband transitions, require high-quality quantum wells and operate only at low temperatures^10^. Terahertz field-effect transistors (FETs)^9,11,12^ usually possess a sub-micrometer scale channel and require a DC bias between the gate and source. When an electromagnetic LWP beam is coupled by an antenna between the source and gate electrodes, it excites an electron density oscillation, which will generate a driving longitudinal electric field through the channel. A resultant direct current (DC) signal can be measured between the source and drain. Common terahertz photoconductive antennas used in terahertz time domain systems, on the other hand, do not require external bias. The terahertz antenna connected to the contact electrodes of a high-resistivity and ultrashort-carrier-lifetime photoconductor (for example low temperature (LT)-GaAs) absorbs the incident terahertz radiation, which then induces an electric field across the photoconductor contact electrodes. Photo-excited EHPs generated by the pumping of a local femtosecond laser are caused to drift by this field, leading to an output photocurrent proportional to the magnitude of the incident terahertz field^13–15^.

Two-dimensional (2D) materials have also been demonstrated for their ability to detect LWPs^12,16–19^. Among them, graphene-based detectors, including FETs^12^, hot-electron bolometers^18^, and photothermoelectric device^19^, have experienced a development boom. Graphene FET detectors show good detection for radiation at 0.3 THz at room temperature^12^. Bilayer graphene hot-electron bolometer demonstrate comparable sensitivity to but much higher speed than Si bolometers at low temperatures (5 K/10 K)^18^. Graphene terahertz detectors relying on photothermoelectric effect have demonstrated a sensitivity greater than 10 V W^−1^ at room temperature and a full-width at half-maximum pulse response of 110 ps^19^. Moreover, terahertz single-photon detectors^20^ have also been realized at temperatures of *T* < 1 K, demonstrating an extremely low NEP of 10^−22^ W Hz^−1/2^. A novel tunable hot-carrier LWP detector based on hot–cold carrier energy transfer at low temperatures (*T* < 30 K) was also reported^21^, which enabled a very long-wavelength infrared response up to 55 μm (5.5 THz).

Recently, surface plasmon polaritons (SPPs) in subwavelength structures^22^ have been attracting tremendous research efforts, as it has multitudinous applications, including extraordinary optical transmission^23,24^, manipulation of cold atoms^25^, wavelength filtering^26^, plasmonic devices^27^, solar cell energy harvesting^28^, metamaterials^29^, and modern molecular sensing and spectroscopy^30,31^. One of the key properties of SPPs is the capacity to induce non-equilibrium electrons^32–39^. Since the plasma frequencies of metals are generally located in the visible or ultraviolet ranges of the electromagnetic spectrum, it is not possible to achieve intense SPP in metals in the millimeter and terahertz wave ranges. To overcome this problem, spoof SPPs were proposed^40^ and realized^41^ from periodic holes in a metal surface. In addition, SPP can also be excited in some semiconductors^42^. Highly doped silicon^43^ and some narrow band gap semiconductors, such as InSb^44–48^, are excellent plasmonic materials for LWPs, owing to their high electron mobility, low electron density, and small effective mass.

In this work, we propose a strategy for direct detection of LWPs in the millimeter and terahertz wave ranges, based on localized SPP-induced non-equilibrium electrons in an antenna-assisted subwavelength ohmic metal–semiconductor–metal (OMSM) structure. The simulation and experimental results from the antenna-assisted subwavelength OMSM structures made of gold and InSb demonstrate that the proposed structures are indeed able to detect photons in the millimeter and terahertz wave ranges. An NEP of 1.5 × 10^−13^ W Hz^−1/2^ is achieved by a device with a spacing of 10 µm under a DC bias current of 3.5 mA for a beam of 0.151 meV photons at room temperature. The experimental results also indicate that the detection performance can be further improved by optimizing the structure and/or decreasing the operating temperature.

## Results

### Design of subwavelength OMSM structures

**Fig. 1:**
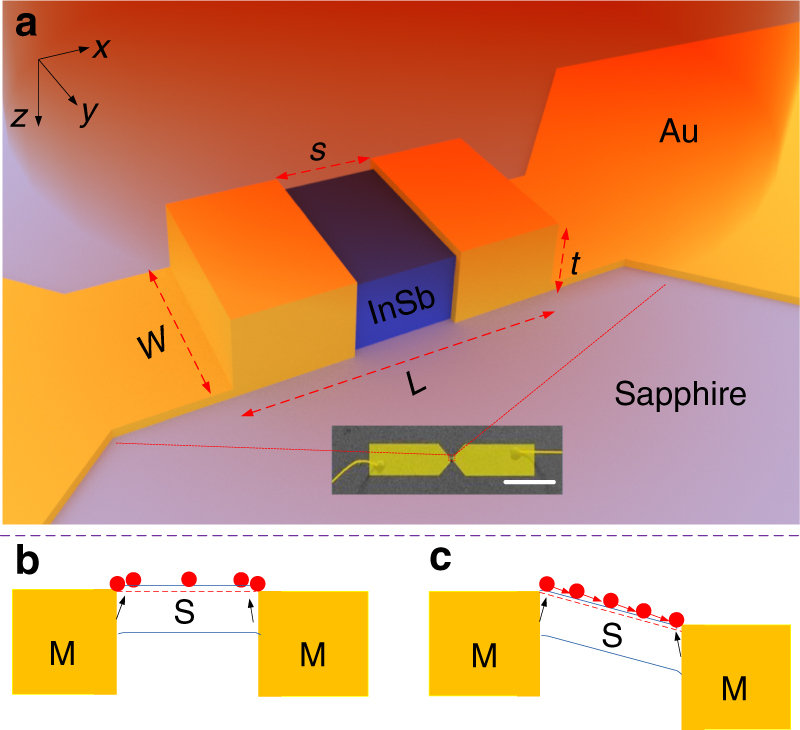
Antenna-assisted subwavelength OMSM structure for millimeter and terahertz waves detection with gold and InSb as metal and semiconductor. **a** Schematic of the ohmic metal–semiconductor–metal (OMSM) structure in the center of the antenna-assisted OMSM structure (not drawn to scale). The wavelength of the incident photons *λ* is much longer than the length of the semiconductor *L* to meet the subwavelength requirement. *s* is the spacing between the edges of the two ohmic contacts. *W* and *t* are the width and thickness of the semiconductor layer, respectively. The inset is a false-colored SEM image of the fabricated device, a planar Au dipole antenna. The scale bar is 1 mm. **b** Zero bias case: symmetry prevents a photocurrent. **c** Biased case: The unidirectional movement of non-equilibrium electrons forms the photocurrent. M and S in **b** and **c** represent metal and semiconductor, respectively

Figure 1a shows the schematic of the proposed antenna-assisted subwavelength OMSM structure, in which the length *L* of the semiconductor and the spacing *s* between the two metallic contacts are much less than the wavelength *λ* of incident waves to be detected. Under transverse magnetic (TM) polarized illumination, the planar dipole antenna efficiently couples photons into the structure, and localized SPPs are excited by the coupled photons within the semiconductor, especially near the semiconductor–metal interfaces on the top facet. The SPPs then induce non-equilibrium electrons by transferring energy to electrons in the semiconductor. With zero bias, the SPP-induced non-equilibrium electrons have a symmetric distribution (Fig. 1b). However, when a bias is applied to the OMSM structure (Fig. 1c), the SPP-induced electrons will flow through the semiconductor, leading to a photocurrent. To allow excitation of localized SPPs in the millimeter and terahertz wave ranges, the semiconductor must have a low plasma frequency (usually with negative permittivity) near the frequency of incident waves, and it should possess high electron mobility to enable fast transit of the SPP-induced non-equilibrium electrons.

We selected InSb as the semiconductor in the OMSM structure as it meets all the requirements. InSb is a well-known III–V semiconductor that is commonly used as an interband transition-based photodetecting material in the infrared range^49^ and in low-temperature thermal hot-electron bolometers based on intraband free electron absorption^50^. The InSb layer used in our experiments has a band gap of 180 meV (Supplementary Fig. 1) and an electron mobility of 5.6 × 10^4^ cm^2^ V^−1^ s^−1^ at room temperature (Supplementary Fig. 2). The plasma frequency of InSb (see Methods and Supplementary Fig. 3) is near 4 THz, corresponding to 16.5 meV, which is in the terahertz wave range. The planar antenna depicted in Fig. 1a is made of gold and is configured as a half-wave dipole. The structure is fabricated on a sapphire substrate.

### Simulations

To gain insight into the excitation of localized SPPs in the proposed structure, we first did numerical simulations using the finite element method (Comsol, RF module). In the millimeter and terahertz wave ranges, the free electrons in the conduction band of InSb behave as a classic solid-state plasma, and the complex dielectric constant is given by Drude model^51,52^. Gold can be regarded as a perfect conductor in the frequency range of interest. The complex dielectric constant of the sapphire substrate was taken from published data^53^.

**Fig. 2:**
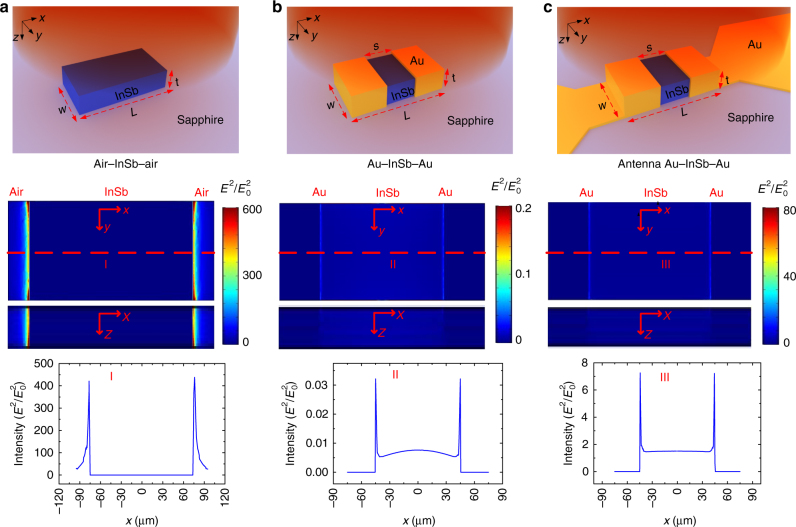
Numerical simulations for the antenna-assisted subwavelength ohmic metal–semiconductor–metal (OMSM) structure made of gold and InSb. Excitation of localized SPPs, the 2D distributions of *E* ^2^/*E*_0_^2^ in *x–y* (*z* = 0) and *x–z* (*y* = 0) planes, and distribution of *E* ^2^/*E*_0_^2^ along the red dash (half width) line for the incident 0.151 meV photons in **a** a bare InSb slab (*L* = 150 μm, *W* = 50 μm, and *t* = 10 μm), **b** the subwavelength Au–InSb–Au structure (the same InSb slab with *s* = 90 μm), and **c** the antenna-assisted subwavelength Au–InSb–Au structure with *s* = 90 μm

We first simulated a single bare InSb slice (air–InSb–air) with a length of 150 μm, a width of 50 μm, and a thickness of 10 μm as shown in Fig. 2a. We selected a TM-polarized (with electric field in *x*-direction) plane wave with a photon energy of 0.151 meV (*λ *= 8 mm) in the simulation as we can experimentally verify the performance at this wavelength. As shown in Fig. 2a, the localized SPP intensity *E* ^2^/*E*_0_^2^ is very strong at the two interfaces between InSb and air but very weak inside the InSb strip. This is understandable as most SPPs should be contained in the dielectric (air in this case). Then, we simulated the subwavelength Au–InSb–Au structure (Fig. 2b) by coating an Au layer on two sides and parts of the top surface of the same InSb strip, separated by a spacing of 90 µm.

It is found that by covering with the perfect conductor (Au), the SPP intensity *E* ^2^/*E*_0_^2^ is still the strongest at the same two edges of the spacing, and it becomes stronger inside the InSb strip than in bare InSb. It should be pointed out that although these primary simulation results for the subwavelength ohmic Au–InSb–Au structures showed excitation of SPPs in the subwavelength Au–InSb–Au structure, the value of *E* ^2^/*E*_0_^2^ is much smaller than one. To enhance the intensity of localized SPPs, we then added a planar Au antenna with a half-wave dipole configuration designed for photons of energy 0.151 meV (see Methods) to the structure. The results for the antenna-assisted structure (Fig. 2c) with the same Au–InSb–Au dimensions still shows a similar SPP distribution, but with intensity enhancement factor of about 310 at the edges and about 200 in the center of the InSb. This is attributable to the antenna’s ability to couple more photons, which, in turn, excite more plasmons in the structure.

**Fig. 3:**
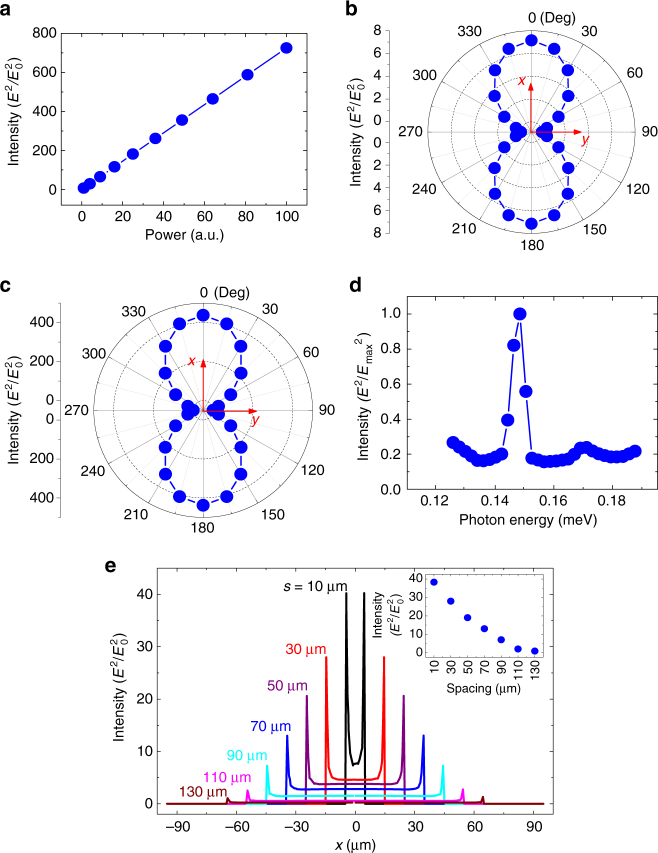
Numerical simulations for the antenna-assisted subwavelength Au–InSb–Au structure. **a** Localized surface plasmon polariton (SPP) intensity (*E* ^2^/*E*_0_^2^) of the structure as a function of incident power. **b** SPP intensity of the structure at different polarization angles for incident 0.151 meV photons. **c** SPP intensity of the air–InSb–air structure at different polarization angles for incident photons of 0.151 meV for comparison. **d** Normalized *E* ^2^/*E*_max_^2^ as a function of incident photon energy. **e** Distribution of *E* ^2^/*E*_0_^2^ along the half width line for devices with spacings *s* of 10, 30, 50, 70, 90, 110, and 130 µm. Inset: *E* ^2^/*E*_0_^2^ as a function of *s* at the point (*s*/2, 0, 0)

For the antenna-assisted subwavelength Au–InSb–Au structure, henceforth the device, we simulated SPP intensities for different powers of the same incident source. The value of *E* ^2^/*E*_0_^2^ at the position (*s*/2, 0, 0) is taken as the representation of SPP intensity. A clear linear relation is observed, as shown in Fig. 3a, as more SPPs will be generated when the number of incident photons increases. Figures 3b, c compare the effects of polarization angle of incident photons on SPP intensity in both the device and the air–InSb–air structure. As shown in Fig. 3c, the SPP intensity in the air–InSb–air structure is sensitive to the polarization angle of the incident light with the maximum and minimum values occurring at the *x* (TM configuration) and *y* (transverse electric (TE) configuration) polarizations, respectively. This is understandable as the TM-polarized light has its electric field perpendicular to the air–InSb interface. For the device, we expect same polarization dependence, as the antenna is designed for coupling more TM-polarized 0.151 meV photons. We then simulated the SPP intensity as a function of incident photon energy. As shown in Fig. 3d, a sharp peak intensity at 0.151 meV confirms that the designed antenna can indeed couple in the most photons at this energy.

We also simulated the localized SPP distributions in devices with fixed *W* and *t* but with different values of spacing *s*. The results, including the *s* = 90 µm device for comparison, are illustrated in Fig. 3e. From the simulations, some phenomena can be observed. First, the maximum SPP intensity always occurs at the two edges of the Au–InSb contacts and increases as spacing decreases. This phenomenon was also been observed in other reports^44,54–56^. Second, SPP intensity is negligible in the gold and decays exponentially with distance inside the InSb strip. Third, the SPP intensity is almost zero in most of the InSb strip when the spacing is large, and it increases as spacing decreases. These observations indicate that the localized SPPs are generated at the two edges of the Au–InSb contacts and decay quickly with distance into the InSb strip. With sufficiently large spacing, the SPPs inside the InSb strip, especially at the center, are negligible and most SPPs exist in the InSb near the two edges. When the spacing is short, however, there are also SPPs throughout the InSb strip, including the center of it. It is believed that the SPPs near the center of the InSb strip are mainly due to the superposition of the SPPs originated from the two edges of the Au–InSb contacts and the superposition should become stronger when the spacing is shorter.

### Experiments

**Fig. 4:**
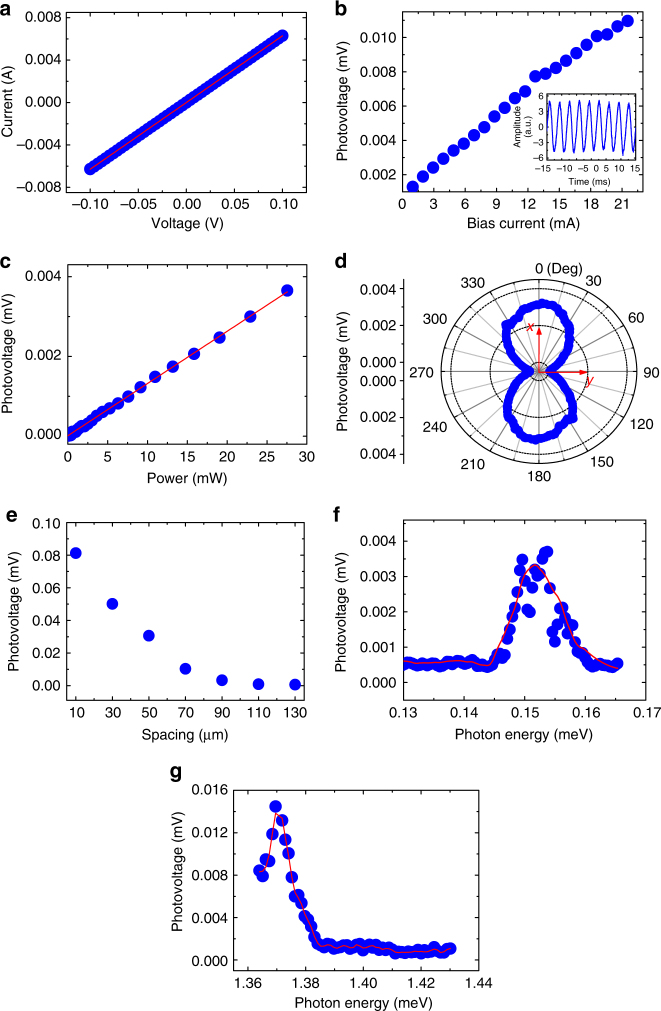
Characterization of the antenna-assisted subwavelength Au–InSb–Au devices. **a** Room temperature current–voltage (*I–V*) characteristics of the device with *s* = 90 µm. The red line is the linear fit to the experimental data. **b** Photovoltage of the same device under 25 mW illumination of a 0.151 meV source at a modulation frequency of 300 Hz, as a function of DC bias current. The inset is a typical photoresponse waveform recorded by an oscilloscope. **c** Photovoltage of the same device as a function of source output power at 300 Hz under a DC bias of 3.5 mA. The red line is the linear fit. **d** Polarization dependence of the photovoltage of the device measured under a DC bias of 3.5 mA and at the source output power of 25 mW. The vertical and horizontal axes are designated as *x* and *y*, respectively. **e** Photovoltages of devices with different values of spacing, measured in the same conditions as for the device with a spacing of 90 µm. **f** Photovoltages of the device for incident waves with photon energies from 0.130 to 0.165 meV under a DC bias of 3.5 mA. The red line is a smoothed version of the data for easier viewing. **g** Photovoltages of the device designed for detecting 1.37 meV (0.332 THz) photons for incident photons from 1.36 to 1.43 meV under a DC bias of 3.5 mA. The red line is a smoothed version of the data

To experimentally verify our new strategy and simulation results, we fabricated a set of antenna-assisted subwavelength ohmic Au–InSb–Au devices, the detailed fabrication process of which can be found in Methods. The first structure tested is the one shown in Fig. 2c, which has an InSb strip with a spacing of 90 µm, a width of 50 µm, and a thickness of 10 µm, and an Au antenna with a length of 4 mm, a width of 0.5 mm, and a thickness of 400 nm. The current–voltage (*I–V*) characteristic of the device in the range of −0.1 to 0.1 V was measured and excellent ohmic behavior was observed (Fig. 4a).

We then characterized photoresponse of the device using the 0.151 meV photon source (Agilent E8257D microwave source combined with a horn, with output powers up to 30 mW), with details shown in Methods. Figure 4b shows the photovoltage as a function of biased DC current, measured with the source output power of 25 mW at a modulation frequency of 300 Hz. The inset illustrates a typical photovoltage waveform recorded by an oscilloscope. Significant photovoltage was indeed observed and it increased with the bias current. The photovoltage is about 0.0032 mV at a bias of 3.5 mA and increases to 0.01 mV at 15 mA. These observations are evidence of direct detection of a millimeter wave.

Photovoltage can be expressed as *V*_ph_ *= rI*_ph_ *= rAn*(*x*)*qμξ*, where *r* is the resistance of the InSb strip, *I*_ph_ is photocurrent, *A* is cross-sectional area of the InSb strip for current flow, *n*(*x*) is the SPP-induced non-equilibrium electron density, *q* is elementary charge, *μ* is electron mobility, and *ξ* is the electrical field associated with the bias. For fixed incident power and at low bias current, all the parameters, including *n*(*x*), which is not uniform in the InSb strip, can be regarded as constants except *ξ* which increases linearly with bias. Hence, the photovoltage increases linearly with biased current due primarily to the increased electrical field^57^ when the bias current is low. When the bias current is more than 12 mA, the photovoltage still increases with bias current but at a slower rate. This is mainly due to the reduced carrier mobility resulting from increased scattering in the semiconductor at high bias.

The photovoltage also shows linear dependence on the source output power in the tested range up to 30 mW (Fig. 4c), which is in excellent agreement with the simulation results shown in Fig. 3a. The linear increase in photovoltage is understandable as more incident photons excite more SPPs, which in turn generate more conduction carriers for photocurrent. By using a calibrated Golay cell, the responsivity of the device obtained at a modulation frequency of 300 Hz under a DC bias current of 3.5 mA is about 50 V W^−1^, corresponding to an NEP of 2.1 × 10^−11^ W Hz^−1/2^ (see Methods). The polarization dependence of the photovoltage for the 0.151 meV source of 25 mW measured at 300 Hz under a DC bias current of 3.5 mA, shown in Fig. 4d, is also similar to the simulated results. That is, the photovoltage is the largest when the polarization is along *x* axis (TM), decreases as the polarization deviates from the *x* axis, and finally disappears when the polarization is along *y* axis (TE).

To study the effect of spacing *s* between the two ohmic contacts on photoresponse, six more ohmic Au–InSb–Au structures (devices) with spacings of 10, 30, 50, 70, 110, and 130 µm but otherwise identical parameters were fabricated and tested under the same conditions as the device of 90 µm spacing. As shown in Fig. 4e, the photovoltage of the devices significantly increases when the spacing decreases. For the device with the minimum (10 µm) spacing, the photovoltage increases to 0.081 mV, about 25 times of that of the 90 µm spacing device, corresponding to a responsivity of 1250 V W^−1^ and an NEP of 1.5 × 10^−13^ W Hz^−1/2^ (see Methods). This observation differs from the trend in traditional photodetectors, where the photovoltage increases with semiconductor area^1^. The discrepancy is justifiable since the semiconductor in traditional photodetectors is the active medium where photogenerated carriers are generated from interband or intraband transitions, whereas the semiconductor InSb strips in the present devices are for excitation of localized SPPs and transit of the SPP-induced non-equilibrium electrons under a bias. In terms of detection performance, a room temperature NEP of 1.5 × 10^−13^ W Hz^−1/2^ is the highest, to the best of our knowledge. A detailed comparison of the device performance between this work and the state-of-the-art is presented in Supplementary Table 1.

To characterize the spectral response of the device, we measured photovoltage using the same source with photon energies varied from 0.130 to 0.165 meV (see Methods). As expected, the photovoltage (in Fig. 4f) shows a sharp peak closed to 0.151 meV as the antenna is designed to couple the most photons into the device at this wavelength. To extend detection to the terahertz wave range, we also designed and fabricated a device which has the same 90 µm spacing but different antenna dimensions for resonance peak at an incident photon energy of 1.371 meV (0.332 THz). As shown in Fig. 4g, the photovoltage measured under 3.5 mA bias indeed exhibits a peak at the expected wavelength with a value of 0.0148 mV for the source output power of 15 mW (VDI source, see Methods), as this is the wavelength of which the maximum number of photons are coupled into the device.

**Fig. 5:**
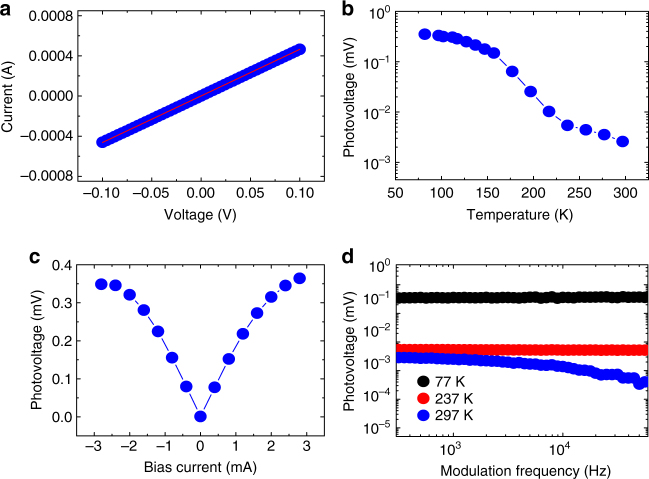
Temperature effects on the performance of the device with *s* = 90 µm. **a** Current–voltage (*I–V*) curve at 77 K. **b** Photovoltage as a function of temperature for source power 25 mW and photon energy 0.151 meV. **c** Photovoltage as a function of bias current at 77 K. **d** Photovoltage–frequency relations at three temperatures. The black, red, and blue dots correspond to 77, 237, and 297 K, respectively

To make our experiment complete, the *s* = 90 µm device was also characterized at temperatures ranging from 77 to 297 K under the same conditions (Supplementary Fig. 4). The 77 K *I–V* curve of the device, as an example, is shown in Fig. 5a and excellent ohmic contact can be observed. Figure 5b shows the photovoltages measured at different temperatures for the 0.151 meV source of 25 mW. The photovoltage increases when the temperature decreases, and the value at 77 K under a bias of 3 mA is about 0.35 mV, which is about 136 times the 0.00257 mV achieved at room temperature. The corresponding responsivity and NEP at this temperature are 5830 V W^−1^ and 1.3 × 10^−12^ W Hz^−1/2^, respectively (see Methods). The NEP as a function of bias current for the device at room temperature and at 77 K are shown in Supplementary Fig. 5a, b. We also show the NEP values of the devices with different spacing, measured under 3.5 mA bias at room temperature, in Supplementary Fig. 5c.

The higher values of photovoltage at 77 K than at room temperature are mainly due to the increased electron mobility of the SPP-induced non-equilibrium electrons as lattice scattering decreases. The 77 K photovoltage versus DC bias current is illustrated in Fig. 5c. It is observed that the photovoltage nearly saturates at about 3 mA due to the saturation of electron velocity under large electric field. At this temperature, the resistance of the device is 250 Ω as derived from Fig. 5a, and the voltage applied to the 90 µm InSb is close to 1 V, corresponding to an electric field of 110 V cm^−1^ which is consistent with the reported value^58^. With increasing temperature, the required electrical field for saturation will be higher^59^.

We also studied the effect of modulation frequency on photoresponse of the device at temperatures from 77 to 297 K and the results obtained at three different temperatures are illustrated in Fig. 5d. It is clearly seen that the photovoltage values are nearly unchanged in the measured frequency range of 10^5^ Hz at lower temperatures, demonstrating a fast response. The photoresponse speed is comparable to traditional photodetectors^1^ and much faster than the thermal Golay cell detectors in the millimeter wave range. At room temperature, however, not only is the photovoltage smaller, but it also decreases when the modulation frequency becomes high. The lowered mobility at room temperature is likely the main factor for these observations as it results in a lower response. It is more difficult for the SPP-induced electrons to follow at an increased modulation frequency, leading to a decrease in photovoltage.

## Discussion

In this paper, we proposed antenna-assisted subwavelength OMSM structures for direct detection of LWPs in the millimeter and terahertz wave ranges via localized SPP-induced non-equilibrium electrons in a low-plasma-frequency and high-electron-mobility semiconductor strip of subwavelength dimension. The devices, made of gold and InSb, were fabricated to verify the new strategy. The antenna is employed to increase the coupling of incident photons into the subwavelength Au–InSb–Au structure, which is used to excite SPPs that generate non-equilibrium electrons in the InSb strip. When a DC bias is applied, the non-equilibrium electrons will flow to form a photocurrent. For the device (with a spacing of 90 µm) designed for detecting 8 mm wave, the responsivity measured at room temperature is about 50 V W^−1^, corresponding to an NEP of 2.1 × 10^−11^ W Hz^−1/2^. By decreasing the spacing to 10 µm, a room temperature NEP of 1.5 × 10^−13^ W Hz^−1/2^ is achieved. The detection performance can be further improved by optimizing the structure dimensions and materials used in the antenna-assisted subwavelength OMSM structures. One can also design devices for detection of any arbitrary wavelength in the millimeter and terahertz wave ranges. In addition, the proposed devices are easy to fabricate and operate. It is believed that this work will open a new avenue for LWP detection and the proposed strategy can be extended to other photonic devices.

## Methods

### Device fabrication

We used single crystal undoped InSb (111) material for the subwavelength OMSM devices. First, the InSb wafer was transferred and affixed onto a sapphire substrate by epoxy glue. It was then polished into a 10-µm-thick film. Using conventional photolithography and chemical solution etching (HF:HAC:H_2_O_2_), a series of strips with a width of 50 µm and a thickness of 10 µm but with varying lengths were formed. Next, the metallic contacts and dipole antennas were defined via photolithography, e-beam evaporation, and a standard lift-off process to form antenna-assisted subwavelength OMSM structures with spacings from 10 to 130 µm. A thickness of 400 nm of Au, following a 15 nm Cr adhesion layer, was deposited to form the ohmic contact and coupling antenna.

### Performance characterization

For the responsivity measurement, the incident radiation was mechanically or electrically modulated, depending on the modulation frequency. The detector under test was mounted in a low-temperature dewar and biased by a direct current. The photovoltage data were collected by an lock-in amplifier or an Oscilloscope after a preamplifier. An Agilent E8257D microwave source combined with a horn was used as the radiation source for photons of 0.130–0.165 meV, and a VDI WR2.2SGX source was used for photons from 1.36 to 1.43 meV. A Golay cell was used to calibrate the responsivity as *R* = *V*/(*pA*) = *VA*_G_ *R*_G_/(*V*_G_ *A*) where *p* is the power density, *V* and *V*_G_ are the output voltage of the OMSM device and Golay cell, respectively, *A* is the effective absorption area of the antenna of the device, described as^15^ *A* = *Gλ* ^2^/(4*π*) (*G* is the gain of the antenna), *A*_G_ is the absorption area of the Golay cell (50 mm^2^), and *R*_G_ is the responsivity of the Golay cell (10^5^ V W^−1^ at 15 Hz). For the *s* = 90 µm device, the output voltage of the Golay cell for 0.151 meV incident photons was 34.5 mV, and the calibrated *p* was 0.69 µW cm^−2^. The output photovoltage under a bias of 3.5 mA was 0.0032 mV, and the *G* calculated by HFSS was 1.73, which give a responsivity of 50 V W^−1^.

From the perspective of real applications, the figure of merit utilized to evaluate the performance of a detector is the NEP, which corresponds to the lowest detectable power in a 1 Hz bandwidth. The lower the NEP, the better the performance of the detector. NEP can be expressed as NEP = *v*_n_/*R*, where *v*_n_ is the root mean square (RMS) of the noise voltage and *R* is the voltage responsivity of the detector. For our detectors, in addition to the thermal Johnson–Nyquist noise (*v*_t_), which usually dominates in terahertz FETs^12^, the noise (*v*_b_) due to bias (dark current) should also be included. The total noise can be described by^1^ *v*_n_ = (*v*_t_^2^ + *v*_b_^2^)^1/2^ = (4*k*_B_ *Tr* + 2*qI*_d_ *r* ^2^)^1/2^, where *k*_B_ is Boltzmann’s constant in joules per kelvin, *T* is the detector’s absolute temperature in kelvin, *r* is the resistance value of the device in ohms (Ω), *q* is the elementary charge, and *I*_d_ is the dark current of the device (bias current in our case).

### Numerical simulations

In the simulations, the permittivity of InSb^60,61^ in the millimeter and terahertz wave ranges can be described by the Drude model *ε*(*ω*) = *ε*_∞_ *ε*_0_[1-*ω*_p_^2^/(*ω* ^2^ + i*ωω*_τ_)], where *ε*_∞_ is the high-frequency permittivity and *ω*_τ_ is the average collision rate of the charge carriers. The plasma frequency *ω*_p_ is defined as *ω*_p_^2^ = *q* ^2^ *n*/(*m* ^*^ *ε*_∞_ *ε*_0_), where *q* is the elementary charge, *n* is the electron density, and *m* ^*^ is the effective electron mass. We also used HFSS to calculate the gain *G* of the antenna in the calibration.

### Data availability

The data that support the findings of this study are available from the corresponding authors upon request.

## Electronic supplementary material

Supplementary Information
