# Supplementary material for: Surface plasmon induced direct detection of long wavelength photons
Source: Nat Commun. 2017 Nov 21;8:1660. doi: 10.1038/s41467-017-01828-2 (PMC5698436; doi:10.1038/s41467-017-01828-2)
Supplement: Supplementary file 1 — Supplementary Information [file 41467_2017_1828_MOESM1_ESM.pdf]

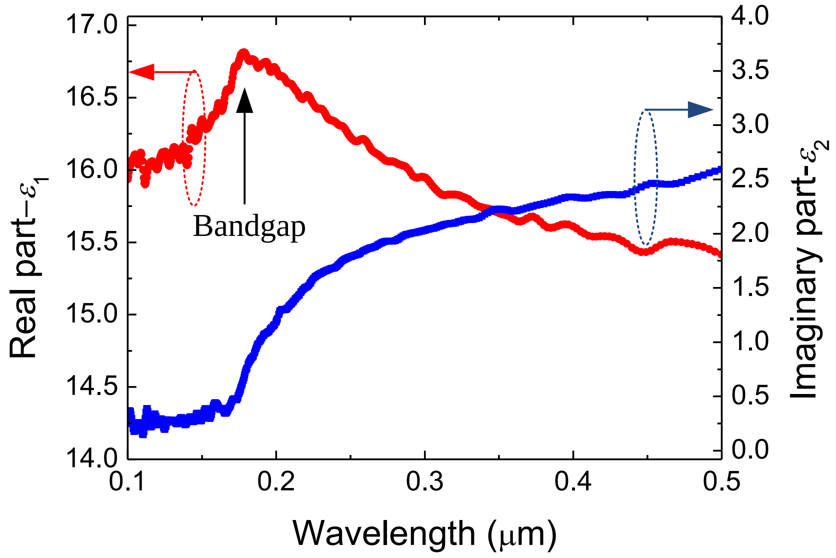

Supplementary Fig. 1 Ellipsometric measurements for the InSb film. The deduced bandgap is 180 meV from the peak position of ε_1. The red and blue lines indicate the real and imaginary part of the permittivity, respectively.

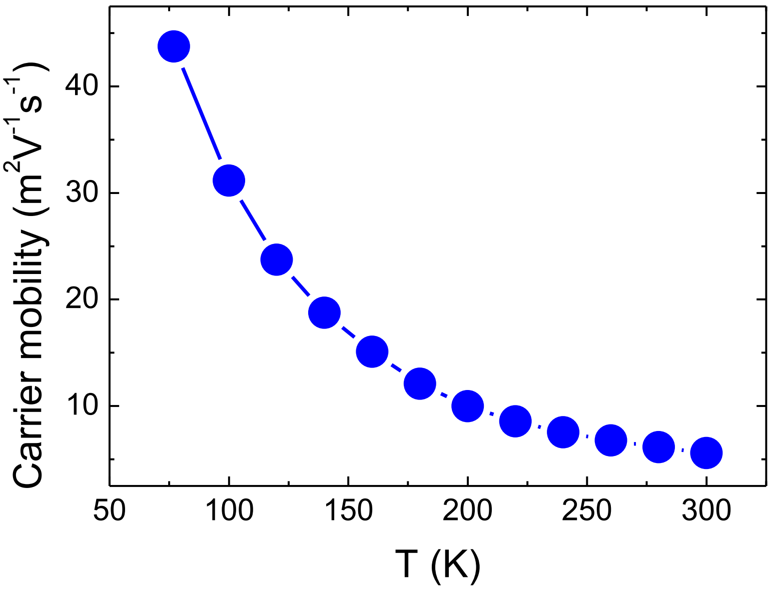

Supplementary Fig. 2 Electron mobilities of InSb obtained from Hall measurements from77 K to 300 K.

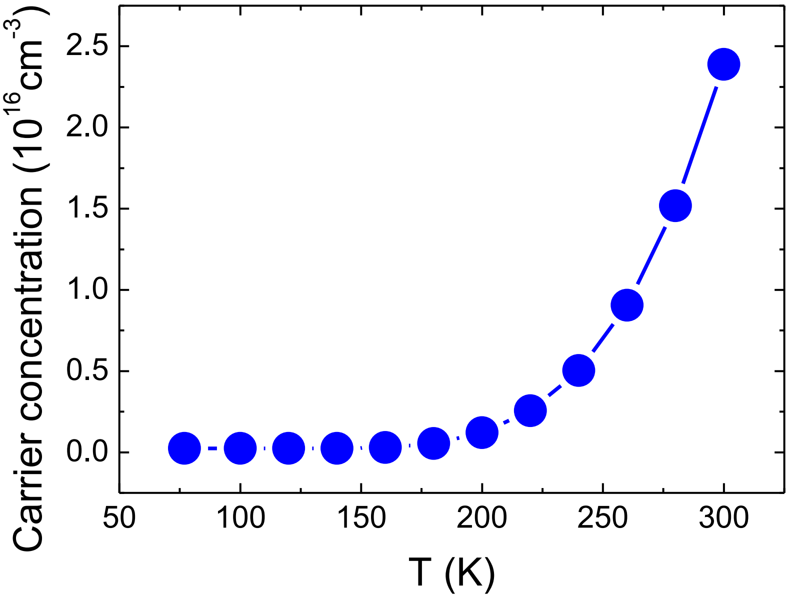

Supplementary Fig. 3 Electron concentrations of InSb obtained from Hall measurements from 77 K to 300 K.

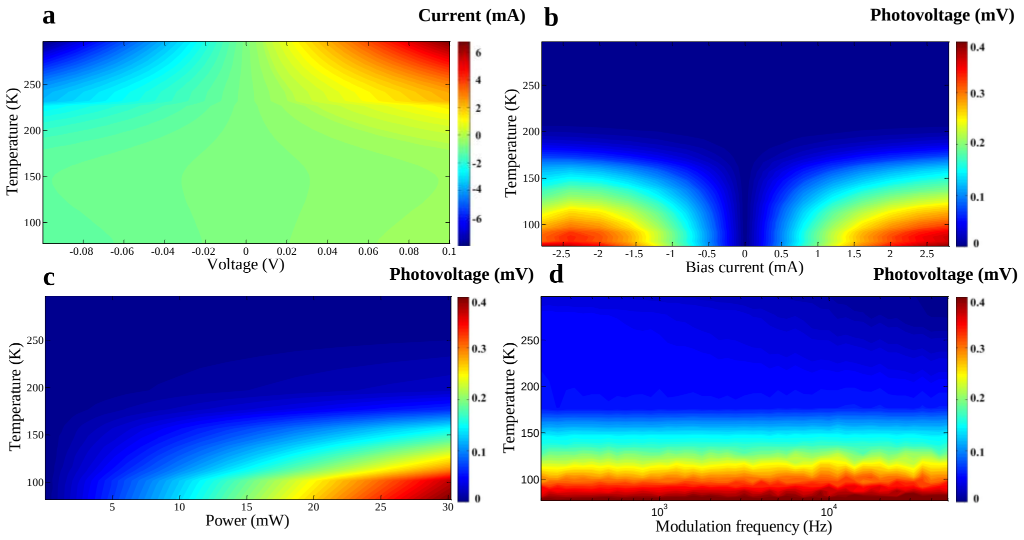

Supplementary Fig. 4 Two-dimensional performance maps of *s*=90 μm device measured at temperatures ranging from 77 to 293 K. (**a**) Current-voltage (I-V) characteristics. (**b**) Photovoltage versus bias current. (**c**) Photovoltage versus output power of source. (**d**) Photovoltage versus modulation frequency.

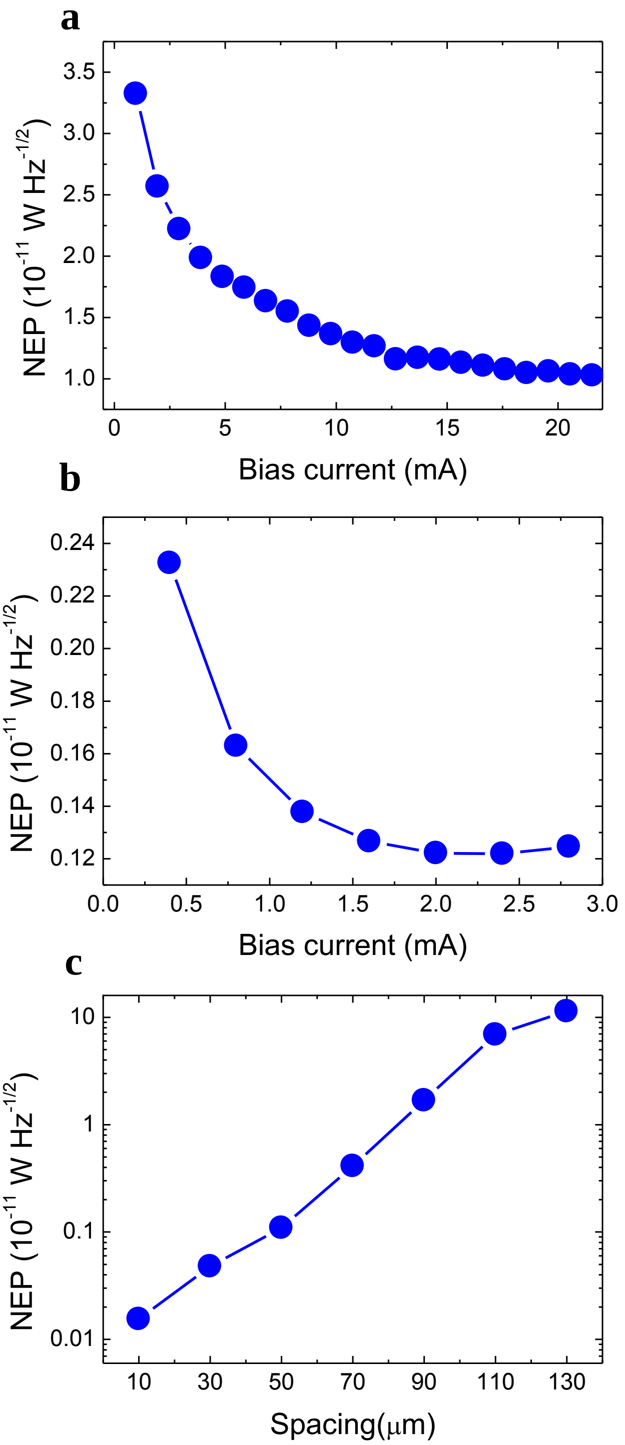

Supplementary Fig. 5 Noise equivalent power (NEP) of the devices. (**a**) NEP of the device with *s*=90 μm at different bias currents at room temperature. (**b**) NEP of the device with *s*=90 μm at different bias currents at 77 K. (**c**) NEP of the device with different spacing values at 3.5 mA at room temperature.

Devices	Mechanism	Typical NEP (W Hz$^{-1/2}$)	Operation range (THz)	Operating temperature	Respond time	Ref
Golay cells (Commercial)	Thermal	10^{-10}-10^{-9}	0.02-20	Room temperature	25 ms	1,2
Piroelectric(Commercial)	Thermal	10^{-9}	0.1-3	Room temperature	10 ms	1
Titanium Nanobolometer	bolometer	10^{-21}	<0.59	<200 mK	-	3
Schottky diodes	Nonlinear	10^{-10} 10^{-12} (no incident power)	0.06-1.5 (depends on antenna)	Room temperature	<ns	4,5
Si FET	Plasma waves in channel	10^{-10}	<0.7	Room temperature	-	6
Si CMOS	CMOS	10^{-11}	-	Room temperature	-	7
Photoconductive antenna	Photocarriers excitation	-	0.1-1.5 (depends on antenna)	Room temperature	<ns	8,9
Graphene	Plasma waves in channel	10^{-9}-10^{-8}	0.3	Room temperature	-	10
Graphene	Hot-electron bolometer	10^{-14}	28.3	5K	ns	11
Graphene	Photothermoelectric effect	10^{-11}	2.52	Room temperature	<ns	12
Black Phosphorus	Plasma waves in channel	10^{-8}	0.3	Room temperature	-	13
Terahertz quantum-well photodetector	QW	-	3.2, 5.4, 9.7	<20 K	-	14, 15
GaAs based devices	Hot holes	-	15	<30 K	<ns	16
Single photon	GaAs/AlGaAs Quantum dot (QD)	10^{-22}	1.43-1.71	0.05 K	ms	17
This work	Surface plasmon induced non-equilibrium electrons	10^{-11} (293 K, 90 μm); 10^{-13} (293 K, 10 μm)	0.01-1 (depends on antenna)	Room temperature and low temperatures	μs (room temperature) ns (77 K)	

Supplementary Table 1 Comparison between our devices and the state-of-the-art.

Supplementary References

1. Sizov, F. & Rogalski, A. THz detectors. *Prog. Quantum Electron.* **34**, 278–347 (2010).
2. Golay Cells Datasheet. http://www.tydexoptics.com/pdf/Golay_Detectors.pdf. Models GC-1P/T/D. Accessed October 18, 2017.
3. Jian, W. et al. Ultrasensitive hot-electron nanobolometers for terahertz astrophysics, *Nature Nanotechnology* **3**, 496 - 500 (2008).
4. Semenov, A. *et al*. Application of zero-bias quasi-optical schottky-diode detectors for monitoring shot-pulse and weak terahertz radiation. *IEEE Electron Device Letters*. **31,** 7 (2010).
5. Mehdi, I. et al. *THz local oscillator sources* (the Far-IR, Sub-mm & mm Detector Technology Workshop, Monterey, CA, 2002).
6. Tauk, R. et al. Plasma wave detection of terahertz radiation by silicon field effects transistors: Responsivity and noise equivalent power, *Appl Phys Lett* **89**, 253511 (2006).
7. Pfeiffer, U. R & Ojefors, E. Terahertz imaging with CMOS/BiCMOS process technologies, *36th European solid-state circuits Conference*. 13–17, 09 (2010).
8. Peng, K. et al, Single Nanowire Photoconductive Terahertz Detectors, *Nano Lett*. **15**, 206–210 (2015).
9. Castro-Camus, E. et al., Polarization-sensitive terahertz detection by multicontact photoconductive receivers, *Appl. Phys. Lett.* **86**, 254102 (2005).
10. Vicarelli, L. et al., Graphene field-effect transistors as room-temperature terahertz detectors, *Nat. Mater*, **11**, 865-871 (2012).
11. Yan, J. et al. Dual-gated bilayer graphene hot-electron bolometer, *Nat. Nanotech.* **7**, 472-478 (2012).
12. Cai, X. H et al., Sensitive room-temperature terahertz detection via the photothermoelectric effect in graphene, *Nat. Nanotech.* **9**, 814-819 (2014).
13. Leonardo, V. *et al*., Black Phosphorus Terahertz Photodetectors, *Adv. Mater.* **27**, 5567–5572. 2015.
14. Liu, H. C., Song, C. Y., Spring, A. J. & Cao, J. C. Terahertz quantum-well-photodetector, *Appl. Phys. Lett.* **84**, 4068-4070 (2004).
15. Luo, H., Liu, H.C., Song, C. Y. & Wasilewski, Z. R. Backgound-limited terahertz quantum-well photodetector, *Appl. Phys. Lett.* **86**, 231103 (2005).
16. Lao, Yan-Feng. et al., Tunable hot-carrier photodetection beyond the bandgap spectral limit, *Nature Photonics* **8**, 412–418 (2014).
17. Komiyama, S., Astafiev, O., Antonov, V., Kutsuwa, T. & Hirai, H. A single-photon detector in the far-infrared range, *Nature* **403**, 405-407 (2000).
